# Cone-beam computed tomography evaluation of the maxillofacial features of patients with unilateral temporomandibular joint ankylosis undergoing condylar reconstruction with an autogenous coronoid process graft

**DOI:** 10.1371/journal.pone.0173142

**Published:** 2017-03-03

**Authors:** Li Liu, Jiayang Li, Huanzhong Ji, Nian Zhang, Yiyao Wang, Guangning Zheng, Hu Wang, En Luo

**Affiliations:** 1 State Key Laboratory of Oral Disease and National Clinical Research Center for Oral Disease, Department of Oral Radiology, West China Hospital of Stomatology, Sichuan University, Chengdu, China; 2 State Key Laboratory of Oral Disease and National Clinical Research Center for Oral Disease, Department of Oral and Maxillofacial Surgery, West China Hospital of Stomatology, Sichuan University, Chengdu, China; Medical University of South Carolina, UNITED STATES

## Abstract

**Objective:**

To evaluate the changes in the jaws and the upper airways of unilateral temporomandibular joint ankylosis patients who underwent condylar reconstruction via autogenous coronoid process grafts using cone-beam computed tomography (CBCT).

**Study design:**

The 27 included patients underwent CBCT examinations at three stages: T0 (within two weeks before surgery), T1 (two weeks after surgery), and T2 (an average of 13 months after surgery). Forty items related to the maxillofacial hard tissues and the upper airway collected at the three times and the coronoid process graft volumes after surgery were compared.

**Results:**

Some integral items related to the mandibular hard tissues exhibited statistical difference shortly after surgery. Some integral items related to maxillofacial hard tissues changing obviously long period after surgery may result from graft remodeling. Asymmetry-related item regarding local neo-condyle and some airway items were significantly different between T0 and T1. Due to variations in graft remodeling, some related local asymmetry items and airway items differed significantly between T0 and T2.

**Conclusions:**

Anteriorly and inferiorly located neo-condyles and a trend toward the pronation of the mandible were observed and the narrowness of the upper airway was improved shortly after surgery. The grafts remodeled differently and some integral and asymmetry items related to neo-condyle changed. The improvements in the upper airway were slightly reduced.

## Introduction

The temporomandibular joint (TMJ) is the only diarthrodial joint with bilateral linkage and is involved in breathing, chewing, swallowing, language, expression, etc. Temporomandibular joint ankylosis is primarily caused by trauma or infection and leads to limitations in mouth opening and corresponding dysfunctions. Moreover, if the ankylosis occurs during development, it may be accompanied by maxillofacial deformities and obstructive sleep apnea syndrome (OSAS), and these conditions strongly influence the patient’s life.

The coronoid process is often elongated in patients with TMJ ankylosis [1], which is associated with reducing mouth opening that needs to be resected and discharged. Free grafting of the autogenous coronoid process for TMJ reconstruction has been used since 1980 [2], and it is thought that the rate of ankylosis recurrence of this procedure is the lowest among several surgical potential surgical procedures.

Some animal studies [3,4] has suggested that condylar reconstruction with an autogenous coronoid process can result in morphology and histology that are similar to those of the normal condyle after adaptive remodeling and seemed not to affect TMJ function or mandibular growth. Clinical research [5] has also revealed satisfactory postoperative results. However, these clinical data have primarily been focused on mouth opening and recurrence and contain limited detail regarding changes in maxillofacial deformities, occlusion, the upper airway and graft remodeling. In this study, we used CBCT images to analyze the maxillofacial deformity of 27 unilateral temporomandibular joint ankylosis patients who received autogenous coronoid process graft reconstruction treatment, to evaluate the changes in the maxillofacial hard tissues and the upper airway, and to assess post-surgical outcomes and stability.

## Materials and methods

The subjects included 27 adults (12 men, 15women; mean age 28.1±9.75 years; range 18–48 years) who had been diagnosed with unilateral skeletal ankylosis in the TMJ that occurred before adolescence and who planned to undergo autogenous coronoid process graft reconstruction treatment. Patients with ankylosis caused by systemic disease and relapsed patients were excluded. The project was approved by the Scientific and Ethics Committee of Sichuan University. The approval number is WCHSIRB-D-2012-091. And all of the participants provide their written informed consent in Chinese to participate in this study.

### CBCT examinations

CBCT (MCT-1, J Morita Mfg. Corp., Kyoto, Kyoto-fu, Japan) scans and 3D reconstructions were performed at three time points: T0 (within two weeks before surgery), T1 (two weeks after surgery), and T2 (an average of 13 months after surgery). The patients were seated with the FH line and horizontal line parallel and in the intercuspal position and instructed to relax the tongue and avoid swallowing while the CBCT images were collected (slice thickness: 0.5mm; tube voltage: 85kV; tube current: 4.0mA; scanning range: the supraorbital area to the chin). Imaging Dolphin Version 11.7 (Dolphin Imaging and Management Solution, Chatsworth, Calif., USA) was used to read the DICOM data and to reconstruct the 3D maxillofacial hard tissues.

### Maxillofacial hard tissue and upper airway measurements

**Fig 1:**
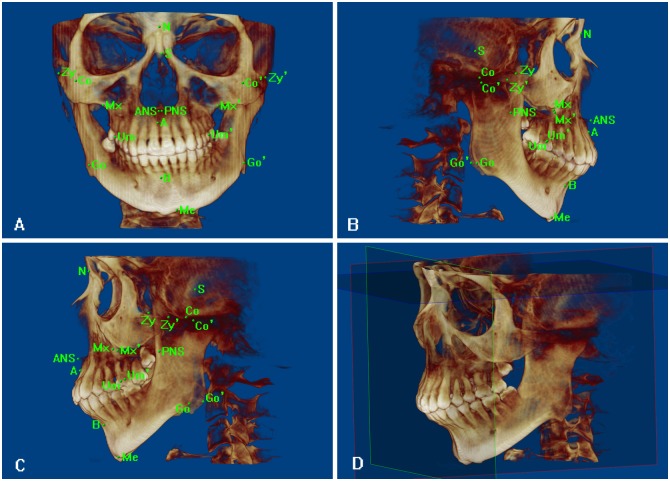
Maxillofacial hard tissue measurements. Relevant points and planes (ABC): N: the most concave and supreme point of nasofrontal suture; S: the center point of the sella turcica; Zy: the most lateral and supreme point of the zygomatic arch divided into Zy (i.e., the healthy side) and Zy’(i.e., the affected side); Co: the supreme point of the mandibular condyle divided into Co (i.e., the healthy side) and Co’ (i.e., the affected side); ANS: the point of the anterior nasal spine; Mx: the most interior and supreme point between the infrazygoma and the maxillary molar divided into Mx (i.e., the healthy side) and Mx’ (i.e., the affected side); PNS: the posterior-most point of the hard palate; A: the most concave point between the anterior nasal spine and the upper alveolar margin sagittally; Um: the most lateral point of first upper molar divided into Um (i.e., the healthy side) and Um’(i.e., the affected side); B: the most concave point of the anterior alveolar bone around lower incisors sagittally; Go: the posterior-most and nethermost point of the mandibular angle divided into Go (i.e., the healthy side) and Go’(i.e., the affected side); Me: the gnathion; Occ: the occlusal plane passing the two centers of the bilateral first upper molar overbite and the center of the central incisors overbite; MP: the plane passing Me, Go and Go’. Coordinate system (D):X: The horizontal plane crossing the straight line that rotated around N 7 degrees upward along NS and paralleled the two innermost points of the zygomaticofrontal suture on both sides; Y: The sagittal plane crossing N and the center point of the crista galli and perpendicular to X; Z: The coronal plane crossing N and perpendicular to X and Y. Fourteen integral items: SNA angle; SNB angle; ANB angle; Occ/X: the minor angle between Occ and X; MP/X: the minor angle between MP and X; ANS-PNS: the distance between ANS and PNS; N-Me(Y): the distance between N and the point that was projected by Me vertically on Z; N-ANS(Y): the distance between N and the point that was projected by ANS vertically on Z; ANS-Me(Y): the distance between two points that were projected by ANS and Me vertically on Z; Zy-Zy’/Y: the lower angle on tonic side between the line crossing Zy and Zy’ and Y; Mx-Mx’/Y: the lower angle on the tonic side between the line crossing Mx and Mx’ and Y; Go-Go’/Y: the lower angle on the tonic side between the line crossing Go and Go’ and Y; Occ/Y: the lower angle on tonic side between Occ and Y; Me(X): the vertical distance between Me and Y. Twelve asymmetrical items: Co-Me: the distance between Co and Me divided into Co-Me and Co’-Me; Go-Me: the distance between Go and Me divided into Go-Me and Go’-Me; Co-Go: the distance between Co and Go divided into Co-Go and Co’-Go’; Go-Me(Y): the distance between the two points that were projected by Go and Me vertically on Y and divided into Go-Me(Y) and Go’-Me(Y); Go(X): the vertical distance between Go and Y divided into Go(X) and Go’(X); Go(Y): the vertical distance between Go and X divided into Go(Y) and Go’(Y); Go(Z): the vertical distance between Go and Z divided into Go(Z) and Go’(Z); Co(X): the vertical distance between Co and Y divided into Co(X) and Co’(X); Co(Y): the vertical distance between Co and X divided into Co(Y) and Co’(Y); Co(Z): the vertical distance between Co and Z divided into Co(Z) and Co’(Z); Co-Go-Me: the angle consisting of Co, Go and Me divided into Co-Go-Me and Co’-Go’-Me; Um-Mx(Y): the distance between two points that were projected by Um and Mx vertically on Y and divided into Um-Mx(Y) and Um’-Mx’(Y).

**Fig 2:**
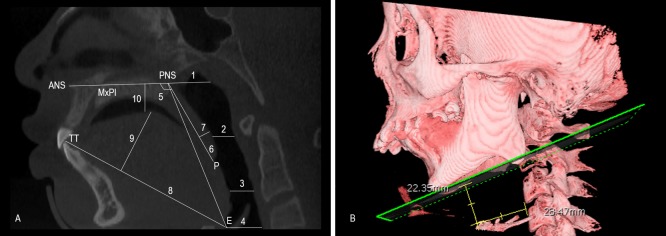
Upper airway measurements. Relevant 2D points and 10 items related to the midsagittal plane (A):ANS; PNS; ut; P; TT; E; MxPl; 1 Nasopas; 2 Velo Pasmin; 3 Oropas; 4 Hypopas; 5 SP position; 6 PNS-P; 7 SP thickness; 8 Ton length; 9 Ton height; 10 Ton position [6]. Relevant 3D points and 4 items related to volume (B):AH: the most anterosuperior point of the hyoid bone; MP: the plane passing Me, Go and Go’; VP: the plane crossing the anterior border of C3 and C4 and paralleling its long axis C3 and C4; AH-MP: the distance between AH and MP; AH(Z): the distance between AH and VP; Pasmin area: the smallest area of the cross section of the upper airway; Airway volume: the volume of upper airway between the Nasopas line and Hypopas line.

**Fig 3:**
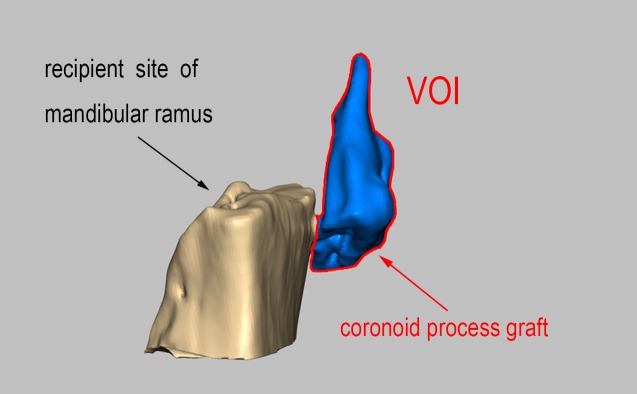
The volume of the coronoid process graft.

In preparation of the analyses, we determined the relevant 3D reference coordinates, planes and points and then measured 14 integral aspects of the maxillofacial hard tissue and 12 asymmetrical factors (Fig 1). Regarding the upper airway, ten 2D items were measured on the midsagittal plane in addition to four 3D items on volume images (Fig 2). The changes in the coronoid process graft volume after surgery were observed at the T1 and T2 points (Fig 3). Differences smaller than 5% in the same patients were not deemed to be obvious changes, decreases between two points greater than 5% were regarded as absorptions, and increases of more than 5% were considered hyperplasia.

### Statistical analysis

If multiple sets of variables were consistent with homogeneity of variance and corresponding normal distribution, the variance analyses of repeated measures were performed with SPSS 19.0 statistical software to compare the 40 items at the three time points. When significant differences were found, the items at two of the time points were compared using post hoc tests to evaluate skeletal stability and upper airway changes. Otherwise Friedman tests and Wilcoxon signed-rank tests were alternative statistical methods. P<0.05 was considered significant.

## Results

All patients treated with condylar reconstruction with an autogenous coronoid process graft tolerated the surgery and achieved satisfactory mouth opening without infection, salivary fistula or facial paralysis in the follow-up period.

### Maxillofacial hard tissue stability

Only the data of three items—the distance between the two points that were projected by Go and Me vertically on Y (Go-Me(Y)), the distance between two points that were projected by Um and Mx vertically on Y (Um-Mx(Y)) and Oropas—missed variance and were compared using Friedman tests and Wilcoxon signed-rank tests. The rest of all items were compared using the variance analyses of repeated measures and post hoc tests.

Among the integral items related to the maxillofacial hard tissues, only some mandibular items, such as SNB angle, ANB angle, the minor angle between Occ and X (Occ/X), the minor angle between MP and X (MP/X), the distance between N and the point that was projected by Me vertically on Z (N-Me(Y)), the distance between two points that were projected by ANS and Me vertically on Z (ANS-Me(Y)), the lower angle on the tonic side between the line crossing Go and Go’ and Y (Go-Go’/Y) and the vertical distance between Me and Y (Me(X)), were significantly different between T0 and T1 (P < 0.05; Table 1). Some integral items related to maxillofacial hard tissues long period after condylar reconstruction via autogenous coronoid process graft exhibited obvious statistical difference, which may result from graft remodeling (P <0.05; Table 1).

**Table 1:** The integral items of craniofacial hard tissue structure at the three time points before and after autogenous coronoid process graft reconstruction for the treatment of unilateral temporomandibular joint ankylosis.

Measurement items	T0	T1	T2
SNA (°)	79.64±3.11	79.75±3.19	78.98±4.01*#
SNB (°)	69.03±4.56	71.21±4.91Δ	70.95±5.07*
ANB (°)	10.61±3.78	8.54±3.92Δ	8.03±4.13*
Occ/X (°)	16.45±6.21	15.83±5.92Δ	16.16±6.18
MP/X (°)	32.17±5.23	33.71±6.91Δ	31.42±7.02#
ANS-PNS (mm)	45.31±3.48	45.12±3.74	44.96±4.01
N-Me(Y) (mm)	111.25±8.52	113.79±8.37Δ	112.15±7.14#
N-ANS(Y) (mm)	52.81±7.52	52.91±8.14	52.73±6.97
ANS-Me(Y) (mm)	58.14±8.56	59.98±7.24Δ	57.41±7.34*#
Zy-Zy/Y (°)	88.11±2.23	88.42±3.12	88.23±2.96
Mx-Mx/Y (°)	92.98±3.34	93.15±3.91	92.63±3.56*#
Go-Go/Y (°)	93.26±2.54	92.13±3.16Δ	93.72±3.15*#
Occ/Y (°)	93.43±3.12	93.52±2.93	92.57±3.17*#
Me(X) (mm)	11.57±4.33	10.03±3.93Δ	11.04±4.81#

^Δ^: T1 compared with T0, P<0.05.

*: T2 compared with T0, P<0.05.

^#^: T1 compared with T2, P<0.05.

**Fig 4:**
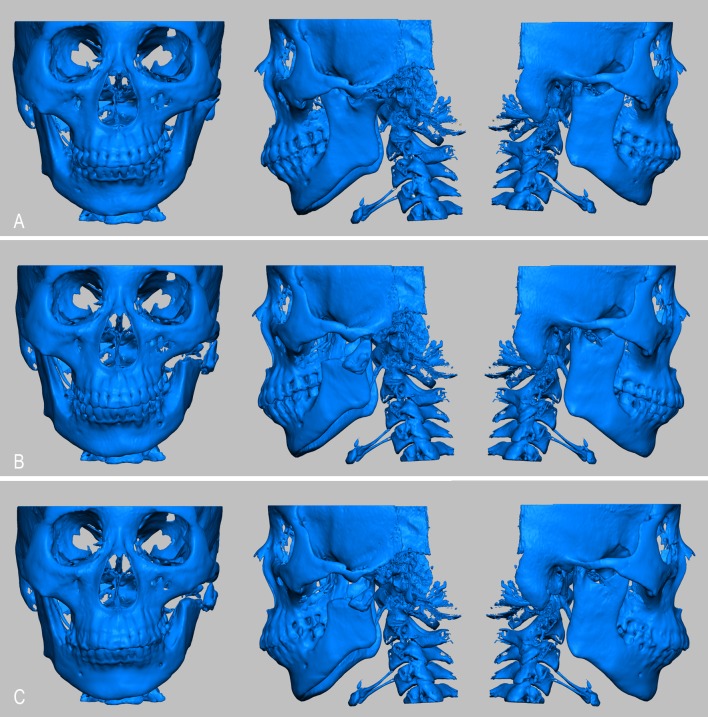
Preoperative maxillofacial hard tissue structures of the autogenous coronoid process graft reconstruction for the treatment of unilateral temporomandibular joint ankylosis at three points (A: T0 point; B: T1 point; C: T2 point).

Regarding the asymmetry items, the length of the affected mandible (Co’-Me’), the length of the affected mandibular ramus (Co’-Go’), the depth of affected mandibular angle (Go’ (Z)), the height and depth of the affected condyle (Co’(Y) and Co’(Z)) and the Co’-Go’-Me angle (Co’-Go’-Me) were significantly different between T0 and T1 (P < 0.05; Table 2). Anteriorly and inferiorly located neo-condyles and a trend toward pronation of the mandible were observed shortly after surgery. Co’-Me’, Co’-Go’, the height of the affected mandibular angle (Go’(Y)), and the height and width of the affected condyle (Co’(Y) and Co’(X)) exhibited significant differences between T1 and T2 (P < 0.05). Co’-Me’, Co’-Go’, Go’(Y), Go’(Z), Co’(Y) and Co’(Z) were significantly different between T0 and T2 (P < 0.05; Table 2). Among the long-term asymmetric items at T2, only those related to local graft remodeling were altered during the follow-up period (Fig 4). For the temporomandibular joint is the only diarthrodial joint with bilateral linkage, some asymmetry items related to health side exhibited significant differences at the three time points (P < 0.05; Table 2, S1 Table).

**Table 2:** The asymmetrical items and their asymmetric indices related to the craniofacial hard tissue structure at the three time points before and after autogenous coronoid process graft reconstruction for the treatment of unilateral temporomandibular joint ankylosis.

Measurement items	T0		T1		T2	
	The affected side	The uninjured side	The affected side	The uninjured side	The affected side	The uninjured side
Co-Me (mm)	81.56±13.42	86.68±12.77	80.12±14.72Δ	87.32±14.75	78.03±13.64*#	87.19±14.93
Go-Me (mm)	52.74±10.63	59.24±9.82	51.84±12.64	60.12±10.73Δ	52.56±11.53	59.36±12.37
Co-Go (mm)	45.64±13.57	53.81±7.49	43.43±12.75Δ	54.84±7.82Δ	41.13±10.36*#	54.32±8.63#
Go-Me(Y) (mm)	36.58±5.06	32.92±4.33	35.97±6.04	32.05±5.84	36.23±5.89	32.42±6.24
Go(Y) (mm)	73.18±5.78	77.01±8.64	72.73±6.04	76.78±6.78	71.12±6.82*#	77.35±7.03#
Go(Z) (mm)	82.65±14.74	82.45±10.43	80.34±13.84Δ	79.73±12.63Δ	80.62±12.37*	80.05±14.29*
Go(X) (mm)	49.77±6.12	41.12±5.89	49.13±7.63	40.63±6.94	49.23±7.25	41.25±7.13#
Co(Y) (mm)	25.17±3.74	17.93±4.12	27.54±4.75Δ	17.42±5.62	29.78±5.23*#	17.35±5.82
Co(X) (mm)	48.94±5.13	50.07±6.12	49.86±7.42	51.04±5.83	49.43±7.14#	51.26±6.27*
Co(Z) (mm)	67.35±9.26	72.6±11.71	61.23±13.62Δ	73.42±10.58	60.68±12.84*	70.57±11.37*#
Co-Go-Me (°)	125.84±6.45	112.53±7.41	123.93±6.93Δ	111.85±7.43Δ	124.13±7.01	112.24±7.26
Um-Mx(Y) (mm)	8.61±2.31	10.09±4.38	9.03±3.03	10.84±3.51	9.41±3.27	11.23±3.72

^Δ^: T1 compared with T0, P<0.05.

*: T2 compared with T0, P<0.05.

^#^: T1 compared with T2, P<0.05.

### Upper airway changes

**Fig 5:**
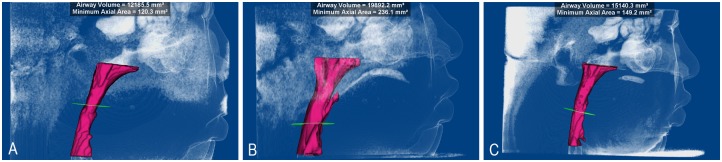
Preoperative upper airways of autogenous coronoid process graft reconstruction for the treatment of unilateral temporomandibular joint ankylosis at three points (A: T0 point; B: T1 point; C: T2 point).

Among the upper airway items, the Velo Pasmin, Oropas, Hypopas, SP position, PNS-P, SP thickness, Ton length, Ton height, Ton position, AH-MP, AH(Z), Pasmin area and Airway volume were significantly different between T0 and T1 (P < 0.05). The narrowness of the upper airway was improved. Among the airway items significant difference in the Nasopas, Velo Pasmin, Oropas, Hypopas, SP position, PNS-P, SP thickness, Ton length, Ton position, AH-MP, AH(Z), Pasmin area and Airway volume were found between T1 and T2 (P < 0.05). Moreover, the Nasopas, Velo Pasmin, Hypopas, SP position, Ton length, Ton position, AH-MP, AH(Z), Pasmin area and Airway volume exhibited significant differences between T0 and T2 (P < 0.05; Table 3, S1 Table). The long-term improvements of the upper airway were slightly reduced at T2 (Fig 5).

**Table 3:** The items related to the upper airway at the three time points before and after autogenous coronoid process graft reconstruction for the treatment of unilateral temporomandibular joint ankylosis.

Measurement items	T0	T1	T2
Nasopas (mm)	20.24±3.89	20.13±4.03	21.12±5.29*#
Velo Pasmin (mm)	2.99±2.41	4.23±2.48Δ	3.63±2.92*#
Oropas (mm)	5.67±2.17	6.89±2.19Δ	6.08±3.15#
Hypopas (mm)	12.31±3.94	12.63±4.37Δ	12.96±4.37*#
SP position (°)	135.74±7.84	133.71±6.83Δ	134.96±7.91*#
PNS-P (°)	43.72±3.83	42.93±4.21Δ	43.83±3.17#
SP thickness (mm)	8.03±1.45	7.52±1.04Δ	7.94±1.16#
Ton length (mm)	78.57±5.82	77.81±6.41Δ	76.38±7.28*#
Ton height (mm)	39.35±5.34	38.93±5.82Δ	38.93±6.16
Ton position (mm)	3.14±2.71	4.27±2.84Δ	3.54±3.01*#
AH-MP (mm)	21.74±5.32	20.74±6.91Δ	22.62±5.14*#
AH(Z) (mm)	31.35±3.79	32.14±4.27Δ	33.72±4.81*#
Pasmin area (mm²)	112.9±98.4	238.5±64.5Δ	173.8±74.2*#
Airway volume (mm³)	13426.9±4163.9	18236.8±5629.5Δ	16428.3±5713.4*#

^Δ^: T1 compared with T0, P<0.05.

*:T2 compared with T0, P<0.05.

^#^: T2 compared with T1, P<0.05.

### Coronoid process graft remodeling

**Fig 6:**
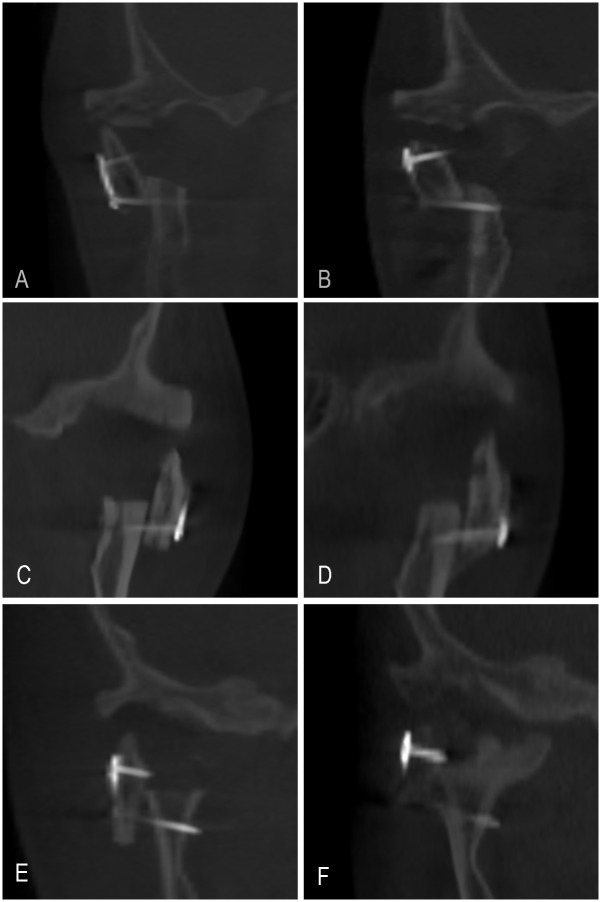
Coronoid process graft remodeling. The coronoid process graft was absorbed, and contact bony formation was not obvious (A: T1 point; B: T2 point). Coronoid process graft absorption was not obvious, and boundary with the new bone tissue was visible (C: T1 point; D: T2 point). coronoid process graft bone hyperplasia with bony fusion (E: T1 point; F: T2 point).

**Fig 7:**
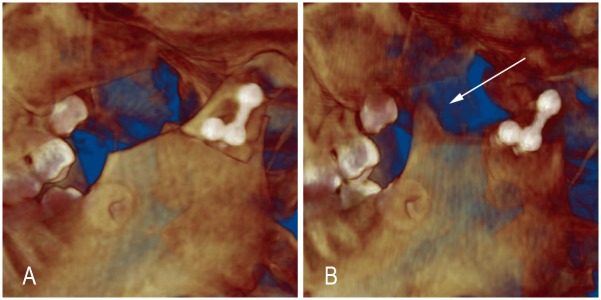
Reduced smaller coronoid regeneration at the former site of the coronoid process resection (white arrow; A: T1 point; B: T2 point).

At T1, all of the autogenous coronoid process grafts were observed to exist with gaps in the newly formed articular fovea. The autogenous coronoid process grafts remodeled differently at T2 (Fig 6). In 16 cases (59.3%), the grafts exhibited absorbed features with flat or rounded bony borders, the volumes of the neo-condyle did not obviously change in 8 cases (29.6%), and in 3 cases (11.1%), the autogenous coronoid process grafts exhibited hyperplasia. Additionally, the connecting parts between the transplanted coronoid process and the mandibular bed exhibited bony fusion in 19 cases (70.4%), in 6 cases (22.2%), new bone tissue was observed at their interface, but the boundary remained visible, and contact bony formations were not obvious in the remaining 2 cases (7.4%). Smaller coronoid bone that regenerated at the former site of the coronoid process resection was observed in 7 cases (25.9%, Fig 7). The remoldings of the coronoid process grafts were not exactly identical, and some integral and asymmetric items related to local remodeling were changing at T1 and T2.

## Discussion

This study demonstrated that some integral items related to the mandibular hard tissues in the unilateral TMA patients changed obviously shortly after condylar reconstruction via autogenous coronoid process graft, and some integral items related to the maxillofacial hard tissues changed at approximately 13 months after surgery, which may result from graft remodeling. Regarding the asymmetry items related to craniofacial hard tissue structure, the length of the affected mandible (Co’-Me’), the length of the affected mandibular ramus (Co’-Go’), the depth of affected mandibular angle (Go’ (Z)), the height and depth of the affected condyle (Co’(Y) and Co’(Z)) and the Co’-Go’-Me angle (Co’-Go’-Me) were significantly different at approximately two weeks after surgery. Anteriorly and inferiorly located neo-condyles and a trend toward pronation of the mandible were observed shortly after surgery, and the maxillofacial deformity could be improved by orthognathic surgery. It is difficult to administer preoperative orthodontic treatment to TMA patients due to limitations of mouth opening. Additionally, TMA patients require early practice with mouth opening to avoid recurrence, but this contradicts intermaxillary fixation for bone union after orthognathic surgery for some time. Therefore, secondary orthognathic surgery is better for maxillofacial malformations caused by temporomandibular joint ankylosis [7]. The autogenous coronoid process grafts remodeled differently in the follow-up period. In 16 cases (59.3%), the grafts exhibited absorbed features with flat or rounded bony borders, the volumes of the neo-condyles did not obviously change in 8 cases (29.6%), and the autogenous coronoid process grafts in 3 cases (11.1%) exhibited hyperplasia. Morphological and structural changes in coronoid process grafts are related to the mechanical environment of the TMJ [8,9]. Some integral and asymmetric items related to the maxillofacial hard tissues changing were observed in our study. The remodeling of the coronoid process grafts may have some effects on the maxillofacial hard tissues. The reason why there is some discrepancy between our research and previous clinical and animal studies [3,5] may be the differences in measurements and statistical methods.

The narrowness of the middle part of the upper airway was improved shortly after surgery, although the patients with unilateral temporomandibular joint ankylosis only received treatment with autogenous coronoid process graft reconstruction. This finding may indicate that this treatment is associated with the relief of ankylosis. The antero-inferiorly located neo-condyle and the pull of the contralateral muscles led to a trend towards the pronation of the mandible long term after surgery. The soft palate is a movable fold of tissue that is suspended from the posterior border of the hard palate and is located above tongue. The position of the soft palate is affected by the location of the mandible location. Muto et al [10] observed that the morphology of the soft palate is associated with tongue base shifts after surgery. Moreover, there is a significant correlation between the morphology of the soft palate and the upper airway volume [11]. These findings are consistent with our findings.

The coronoid process is often elongated in patients with TMJ ankylosis [1], and the consequent reduction in mouth opening requires resectiong and discharging. Free grafting of an autogenous coronoid process for TMJ reconstruction has been used since 1980 [2] and has many advantages: a simple operative technique that does not involve the opening of a second operation area; autogenous grafts prevent transplantation rejection [12]; and this procedure seemed not to affect TMJ function and mandibular growth [3]. However, coronoid process graft absorption has been the focus of clinical debates. Although our research demonstrated that the remodeling of the autogenous coronoid process grafts differed from graft absorption in some cases (16/27) at T2, the reconstructions of the coronoid process grafts may have some effects on the maxillofacial hard tissue.

It is thought that snoring often occurs in TMA patients with maxillary malformations due to varying degrees of obstructive sleep apnea syndrome(OSAS) [13], and snoring affects various systems of the body in increases the risk of death. Previous studies have primarily focused the effects of orthognathic surgery on the upper airway [10,14]. This study found that the narrowness of the middle part of the upper airway was improved two weeks after temporomandibular reconstruction surgery via an autogenous coronoid process graft and that the long-term improvement in the upper airway was slightly reduced.

Zhu et al [3] used a goat model to prove that condylar reconstruction via an autogenous coronoid process results in condylar morphology and histology that are similar to those of the normal condyle after adaptive remodeling and seems not to affect TMJ function or mandibular growth. The remodelings of the goats’ grafts were slightly different from the various changes observed in our research. The differences in the mechanical environment of the TMJ and occlusion between healthy goats and TMA patients may be responsible for these discrepancies. Zhang et al [15] found that the treatment of unilateral TMA with autogenous coronoid process grafts and costochondral grafts for condylar reconstruction were comparable in terms of maximal mouth opening, lateral excursion and mandibular deviation, and these authors speculated that the heights of two grafts were similar in the follow-up period. In contrast, we focused on the changes in the coronoid process graft volume, and the variations in the remodeling of the coronoid process graft might differ.

The long-term improvements in the upper airway were slightly reduced compared with the short-term improvements after surgery, which might be associated with coronoid process graft reconstruction. However, the morphology of the upper airway is influenced by the surrounding structures, including the uvula, lingual root, tonsils, etc. due to the lack of a bony framework. The posterior part of the soft palate is movable and located above tongue base, and its position is affected by respiration, swallowing, posture, etc. Additionally, upper airway obstruction is also influenced by the adhesion of the airway mucosa and the nervous reflex of the inspiratory muscles that is elicited by airway collapse. Therefore, the stability of upper airway improvements in unilateral temporomandibular joint ankylosis patients after condylar reconstruction via autogenous coronoid process grafting needs to be confirmed by further observation.

Compared to 2D plain film radiographs, CBCT does not have the problems of overlap and distortion. Additionally, the radiation dose, pixel isotropy, and display of the hard tissues of CBCT are better than those of traditional spiral CT [16, 17]. Sears CR et al [18] compared upper airways on 2D X-ray film and 3D CBCT images and found correlations between the linear and volumetric measurements. For accuracy and quantitative measurement, the CBCT data was used to build 3D reconstructions in this study. While the limitation of CBCT imaging is the density resolution, the facial soft tissue, which are important for the maxillofacial profile, can be studied further via combinations with other methods.

## Supporting information

S1 Table Most underlying data related to measured items. (XLSX)
